# Physiological-immune resilience risk assessment model for predicting adverse cardiac outcomes in patients with acute myocardial infarction

**DOI:** 10.3389/fcvm.2025.1677614

**Published:** 2026-02-18

**Authors:** Ling Zhang, Qiuyue Wang, Yunying Ji, Chunya Sha, Qiuping Zhang

**Affiliations:** 1 Department of Nursing, Changshu No.2 People's Hospital, Affiliated Changshu Hospital of Nantong University, Changshu, Jiangsu, China; 2 Party Committee Office, Changshu No.2 People's Hospital, Affiliated Changshu Hospital of Nantong University, Changshu, Jiangsu, China; 3 Department of Cardiovascular Medicine, Changshu No.2 People's Hospital, Affiliated Changshu Hospital of Nantong University, Changshu, Jiangsu, China; 4 Department of General Internal Medicine, Changshu No.2 People's Hospital, Affiliated Changshu Hospital of Nantong University, Changshu, Jiangsu, China

**Keywords:** acute myocardial infarction, arrhythmia, high-sensitivity C-reactive protein, lymphocyte, systemic immune-inflammation index, systemic inflammatory response index

## Abstract

**Background:**

Adverse cardiac events have been identified as a major determinant of poor prognosis in patients with acute myocardial infarction (AMI), directly increasing mortality risk. Therefore, this study aimed to establish a physio-immune resilience risk assessment model to identify, at an early stage, the influencing factors of adverse cardiac outcomes in AMI patients, thereby providing clinical guidance for subsequent interventions.

**Methods:**

A total of 345 patients diagnosed with AMI between August 2022 and March 2024 were prospectively enrolled. The occurrence of 30-day major adverse cardiac events (MACE) was independently assessed by two cardiology specialists, and participants were categorized into a MACE group and a non-MACE group accordingly. Clinical data and laboratory findings were compared between groups. Least Absolute Shrinkage and Selection Operator (LASSO) regression and multivariate logistic regression were applied to determine the influencing factors of adverse cardiac outcomes. Furthermore, Receiver Operating Characteristic (ROC) curve analysis was performed to evaluate the predictive value of the physio-immune resilience model for MACE in AMI patients.

**Results:**

The left ventricular ejection fraction (LVEF) in the MACE group was significantly lower than that in the non-MACE group, whereas high-sensitivity C-reactive protein (hs-CRP) levels were markedly higher (*P* < 0.05). The physiological resilience score of the MACE group was lower than that of the non-MACE group, while the European Quality of Life Five-Dimension Scale (EQ-5D) score was significantly higher (*P* < 0.05). Moreover, the lymphocyte count was lower in the MACE group, but both the systemic immune-inflammation index (SII) and the systemic inflammatory response index (SIRI) were higher than those in the non-MACE group (*P* < 0.05). Results from LASSO and multivariate logistic regression indicated that the physiological resilience score (OR = 0.812) served as an independent protective factor for adverse cardiac events in AMI patients, whereas hs-CRP (OR = 1.622), SII (OR = 1.054), and SIRI (OR = 25.905) were independent risk factors. ROC analysis revealed that the combined predictive model incorporating the physiological resilience score, hs-CRP, SII, and SIRI yielded a higher area under the curve (AUC) than any single variable (*P* < 0.05). The model was validated using bootstrap resampling (1,000 iterations), with a Nagelkerke R^2^ = 0.543, suggesting a strong explanatory power for the dependent variable and good calibration performance. The Decision Curve Analysis (DCA) curve was consistently higher than the two extreme curves, indicating greater net clinical benefit of the model-derived predictors.

**Conclusion:**

Patients with AMI undergoing percutaneous coronary intervention (PCI) remain at risk of adverse cardiac outcomes, which may be associated with the physiological resilience score, hs-CRP, SII, and SIRI. These indicators demonstrate substantial predictive value for 30-day MACE in AMI patients. Accordingly, early clinical interventions targeting these risk factors are recommended to reduce the incidence of adverse cardiac outcomes and improve patient prognosis.

## Introduction

Acute myocardial infarction (AMI) has been identified as one of the most impactful non-communicable diseases worldwide, accounting for approximately one-third of all global deaths (1). AMI, a form of unstable ischemic syndrome leading to myocardial necrosis, represents a major contributor to the burden and mortality associated with cardiovascular diseases. It has been estimated that approximately five hundred fifty thousand cases of first-onset AMI and two hundred thousand recurrent cases occur globally each year, comprising nearly half of all cardiovascular disease cases (2). Under usual circumstances, percutaneous coronary intervention (PCI) can effectively improve the prognosis of patients with AMI; however, considerable variability remains, and survival outcomes after the initial PCI procedure show substantial heterogeneity among patients (3). According to previous studies (4), major adverse cardiac events (MACE) in AMI patients, including all-cause mortality, non-fatal AMI, arrhythmias, heart failure readmissions, revascularization, stroke, and major bleeding events, have been closely associated with AMI-related mortality.

Both immune and inflammatory responses play significant roles in the pathogenesis of AMI. Quantifying immune cell balance (5, 6). Immune resilience integrates both immune competence and inflammatory status, effectively reflecting the recovery capacity of the immune system. In patients with AMI, acute rupture of atherosclerotic plaques leads to intense infiltration of neutrophils (NEU), monocytes (MON), and lymphocytes (LYM) into the vascular endothelium. Furthermore, ischemia–reperfusion injury following AMI can amplify the inflammatory cascade, thereby aggravating the condition and promoting the continuous progression of the disease (7). Based on this rationale, specific data integration strategies have been employed to statistically calculate different types of blood cells, leading to the development of novel immune resilience indicators such as the systemic immune-inflammation index (SII) and the systemic inflammatory response index (SIRI). These indices accurately reflect the balance between the body's inflammatory state and immune response, thereby providing a more comprehensive immune evaluation. In addition, the concept of physiological resilience is commonly applied to elderly patients and refers to the body's ability to recover or maintain function when facing age-related damage or disease. A retrospective study demonstrated that factors such as genetics and lifestyle habits could influence the incidence and post-diagnostic survival rate of cardiovascular diseases by modulating physiological resilience, suggesting that physiological resilience may hold predictive value for adverse cardiac outcomes in patients with AMI. In addition, the concept of physiological resilience is commonly applied in older adults and primarily refers to the capacity of the body to recover or maintain function in the face of age-related damage or disease. A high level of physiological resilience can effectively reduce the risk of mortality. A retrospective study found that factors such as genetics and lifestyle habits can influence the incidence of cardiovascular disease and post-diagnosis survival by modulating physiological resilience, suggesting that physiological resilience has predictive value for adverse cardiac outcomes in patients with AMI (8).

In light of these considerations, it is of paramount importance to develop a physiological-immune resilience risk assessment model by integrating measures of physiological resilience (e.g., physiological resilience measurement tools) and immune resilience (e.g., SII, SIRI) to predict adverse cardiac outcomes in patients with AMI. Such a model would allow for a comprehensive evaluation of the physiological and immune resilience status of patients, thereby providing a scientific basis for clinical decision-making. An in-depth investigation was conducted. Building on this premise, the present study conducts an in-depth investigation to enable individualized risk assessment and to inform clinical decision-making.

## Methods

### Research participants

A prospective cohort design was employed. A total of 345 patients diagnosed with AMI between August 2022 and March 2024 were prospectively enrolled. The presence of 30-day MACE was independently assessed by two cardiologists. Based on the occurrence of MACE, patients were categorized into two groups: the MACE group and the non-MACE group. The study was approved by the Hospital Ethics Committee, and informed consent was obtained from all patients and their families.

### Diagnostic criteria

The diagnostic criteria for AMI (9) are as follows: For Type 1 AMI, elevated and/or decreased cardiac troponin (cTn) levels are required, with at least one value exceeding the 99th percentile upper reference limit (URL), accompanied by at least one of the following conditions: a. Presence of symptoms consistent with acute myocardial ischemia; b. Occurrence of new ischemic electrocardiographic (ECG) changes; c. Development of pathological Q waves; d. Imaging evidence of new loss of viable myocardium or new regional wall motion abnormality consistent with an ischemic etiology; e. Identification of a coronary thrombus by angiography or autopsy. For Type 2 AMI, elevated and/or decreased cTn levels are also required, with at least one value above the 99th percentile URL, together with evidence of an imbalance between myocardial oxygen supply and demand unrelated to coronary thrombosis, and at least one of the following criteria must be met: a. Presence of symptoms indicative of acute myocardial ischemia; b. Occurrence of new ischemic ECG changes; c. Development of pathological Q waves; d. Imaging demonstration of new loss of viable myocardium or new regional wall motion abnormality, consistent with an ischemic mechanism.

### Inclusion and exclusion criteria

Inclusion criteria: (1) Age ≥60 years; (2) Diagnosis of an AMI and hospital admission; (3) Time from onset to admission ≤24 h; (4) No prior history of AMI; (5) No contraindications to PCI and undergoing primary PCI for the first time.

Exclusion criteria: (1) The initial diagnosis upon admission was confirmed as chronic coronary syndrome (CCS); (2) Severe peptic ulcer disease, coagulation disorders, severe infections, malignancies, or connective tissue diseases; (3) Cases with incomplete medical records or insufficient short-term follow-up data were excluded; (4) Comorbid pulmonary heart disease or congenital heart disease; (5) Patients with coexisting autoimmune diseases or chronic inflammatory disorders; (6) Patients who had received corticosteroid or immunosuppressive therapy within three months prior to admission.

### General information

General information of the enrolled patients—including gender, age, body mass index (BMI), smoking history, drinking history, history of hypertension, history of diabetes, place of residence, and family history—as well as clinical characteristics such as cardiac function classification, number of diseased vessels, onset-to-admission time, disease type, AMI subtype, and infarction site, were collected by members of the research team through the Hospital Information Management System.

### Grouping method

Follow-up data were obtained through electronic medical record (EMR) review, telephone follow-up, and outpatient interviews. Two cardiology specialists independently determined whether each patient had experienced 30-day MACE. In cases of disagreement, a third cardiology specialist reviewed and adjudicated the case. Based on the final assessment, patients were divided into two groups: the MACE group and the non-MACE group. The types of MACE included all-cause death, non-fatal AMI, arrhythmia [including ventricular fibrillation, sustained ventricular tachycardia (lasting ≥30 s or <30 s with hemodynamic instability), second-degree type II atrioventricular block, third-degree atrioventricular block, and sick sinus syndrome (manifested as marked bradycardia with heart rate <50 bpm accompanied by dizziness or syncope symptoms), re-hospitalization for heart failure, revascularization, and stroke (including both ischemic and hemorrhagic stroke). Ischemic stroke was defined as ① the sudden onset of focal neurological deficits lasting ≥24 h or radiological evidence of new cerebral infarction, and ② exclusion of transient ischemic attack (TIA). Hemorrhagic stroke was defined as confirmed intracerebral or subarachnoid hemorrhage on cranial CT/MRI, with exclusion of trauma, vascular malformation, or other non-AMI-related causes. In addition, major bleeding events were classified as BARC (Bleeding Academic Research Consortium) type 3 or higher, including: ① BARC type 3a – overt bleeding requiring medical intervention; ② BARC type 3b – bleeding associated with a ≥3 g/dl drop in hemoglobin; ③ BARC type 3c – fatal bleeding; ④ BARC type 4 – intracranial hemorrhage; ⑤ BARC type 5 – bleeding leading to death.

### Laboratory indicators

Upon admission, 10 ml of peripheral venous blood was collected from each patient and allowed to stand at room temperature until coagulation occurred. The samples were then centrifuged at a speed of 3,500 r/min, with a radius of 8 cm for 10 min. After centrifugation, the serum was carefully separated and stored for subsequent testing. A fully automated biochemical analyzer (Beckman AU5800) was used to determine creatine kinase–MB (CK-MB) levels, while a fully automated chemiluminescence immunoassay analyzer (Beckman ACCESS 800 microparticle system) was employed to measure cardiac troponin I (cTnI). Detection of high-sensitivity C-reactive protein (hs-CRP) and interleukin-6 (IL-6) was conducted using the enzyme-linked immunosorbent assay (ELISA) method and corresponding reagent kits (Shanghai Jianglai Biotechnology Co., Ltd.), following the manufacturer's instructions. Additionally, cardiac ultrasonography was performed using a Mindray Resona 9 echocardiography system to assess left ventricular ejection fraction (LVEF) and left ventricular end-diastolic volume (LVEDV).

### Physiological resilience score (10)

The Physiological Resilience Index for Older Adults (PRIFOR) was employed as the assessment tool for physiological resilience. This instrument comprises 16 items rated on a 5-point Likert scale (1 = strongly disagree, 5 = strongly agree). The total score is obtained by summing all item scores, with higher total scores indicating greater physiological resilience. The Cronbach's α coefficient of the scale was 0.94, indicating excellent reliability and validity.

### Quality of life assessment

The assessment primarily included evaluations of health-related quality of life, depressive symptoms, and frailty status: ① *Health-related quality of life* was measured using the EuroQol 5-Dimensions (EQ-5D) scale (11), which consists of five dimensions: mobility, self-care, usual activities, pain/discomfort, and anxiety/depression. Each dimension was rated on a three-point Likert scale: 1 = no difficulty, 2 = some difficulty, and 3 = extreme difficulty. The total score ranged from 5 to 15, with lower scores indicating better quality of life. The reliability coefficient of the scale was 0.824, and the test–retest reliability was 0.867, demonstrating good reliability and validity. ② Depressive symptoms were assessed using the 15-item Geriatric Depression Scale (GDS-15) (12), which evaluates patients’ psychological status, emotional experience, life satisfaction, loss of interest, sense of loneliness, helplessness, and concentration ability over the previous week. Each item was answered with “yes  = 1” or “no = 0”, giving a total score range of 0–15, where higher scores indicate more severe depression. The reliability coefficient of the scale was 0.793, and the test–retest reliability was 0.728, reflecting good reliability and validity. ③ Frailty was assessed using the Clinical Frailty Scale (CFS) (13), which evaluates patients’ daily activity ability, functional status, cognition, and perception. Scores range from 1 to 9, with higher scores representing greater frailty. The reliability coefficient of the scale was 0.825, and the test–retest reliability was 0.806, also indicating good reliability and validity.

### Immune resilience indicators

On the morning of the second day after admission, 5 ml of fasting venous blood was collected from each patient into an anticoagulant tube. The samples were centrifuged at a speed of 3,000 r/min, with a radius of 6 cm for 10 min. After centrifugation, the supernatant was carefully collected and stored for subsequent analysis. A fully automated hematology analyzer (Mindray, Nanjing Bede Medical Co., Ltd.) was used to measure NEU, MON, LYM, and platelets (PLT). In addition, derived hematological inflammatory indicators were calculated, including the monocyte-to-lymphocyte ratio (MLR), SII (SII = PLT × NEU/LYM), and systemic inflammation response index (SIRI = NEU × MON/LYM).

### Observational indicators

All patients were categorized according to the presence or absence of 30-day MACE, and the clinical records of the two groups were comparatively analyzed. Laboratory parameters—including CK-MB, cTnI, hs-CRP, IL-6, LVEF, and LVEDV—were analyzed. Additionally, quality of life measures (physiological resilience score, EQ-5D, GDS-15, and CFS) and immune resilience indicators (NEU, MON, LYM, PLT, MLR, SII, and SIRI) were compared between groups.

### Statistical analysis

Statistical analysis was conducted using SPSS version 25.0. Categorical variables were expressed as frequencies and percentages [*n* (%)], and compared using χ^2^ test. Continuous variables were tested for normality using the Shapiro–Wilk method. Normally distributed data were presented as mean ± standard deviation and compared between groups using independent samples *t*-tests; within-group comparisons were conducted using paired samples *t*-tests. Non-normally distributed data were expressed as median [M (P_25_, P_75_)] and analyzed using the non-parametric Mann–Whitney *U*-test. Least absolute shrinkage and selection operator (LASSO) regression and multivariate *logistic* regression were performed to identify factors influencing adverse cardiac outcomes in AMI patients. The significance level was set at *α* = 0.05. Receiver operating characteristic (ROC) curve analysis was subsequently conducted to evaluate the predictive value of the physiological-immune resilience model in assessing adverse cardiac outcomes in AMI patients. A *P*-value <0.05 was considered statistically significant.

## Results

### Comparison of demographic characteristics between the MACE and non-MACE groups

A total of 345 patients with AMI were divided into groups based on the presence or absence of 30-day MACE. Among them, 33 patients experienced MACE, with an incidence rate of 9.57%, including 1 case of all-cause death, 2 cases of non-fatal AMI, 4 cases of arrhythmia, 5 cases of rehospitalization due to heart failure, 2 cases of revascularization, 11 cases of stroke, and 8 cases of major bleeding events. According to statistical analysis, the MACE group included 33 patients, and the non-MACE group included 312 patients. When comparing the demographic characteristics between the two groups, the results showed that the mean age of the MACE group was 69.58 years, which was not significantly different from 70.23 years in the non-MACE group (*P* > 0.05). The BMI of the MACE and non-MACE groups was 23.57 and 23.82 kg/m^2^, respectively, with no significant difference (*P* = 0.377). In the MACE group, 12 patients had a smoking history and 15 had a drinking history, compared with 109 and 115 cases, respectively, in the non-MACE group, showing no significant differences (*P* = 0.870 and 0.333). Additionally, 13 patients in the MACE group had a history of hypertension and 10 had diabetes, compared with 126 and 134 cases in the non-MACE group, respectively, with no significant differences (*P* = 0.912 and 0.161). Furthermore, there were no significant differences between the two groups in terms of sex, place of residence, or family history (*P* = 0.842, 0.064, and 0.616; Table 1).

**Table 1:** Comparison of demographic characteristics between the MACE and non-MACE groups.

Demographic characteristics		MACE group (*n* = 33)	Non-MACE group (*n* = 312)	*χ*^2^/*t*	*P*
Gender	Male	19	174	0.040	0.842
	Female	14	138		
Age ($\bar{\chi }$ ± s, years)		69.58 ± 5.27	70.23 ± 2.01	1.420	0.157
BMI ($\bar{\chi }$ ± s, kg/m^2^)		23.57 ± 1.49	23.82 ± 1.55	0.884	0.377
Smoking history	Yes	12	109	0.027	0.870
	No	21	203		
Drinking history	Yes	15	115	0.939	0.333
	No	18	197		
History of hypertension	Yes	13	126	0.012	0.912
	No	20	186		
History of diabetes	Yes	10	134	1.963	0.161
	No	23	178		
Place of residence	Urban	26	195	3.439	0.064
	Rural	7	117		
Family history	Yes	8	64	0.251	0.616
	No	25	248		

### Comparison of disease-related characteristics between the MACE and non-MACE groups

To analyze the disease-related characteristics of patients in the MACE and non-MACE groups, the following results were obtained: In the MACE group, cardiac function was primarily classified as NYHA Class III–IV in 21 cases (63.64%), which was significantly higher than 124 cases (39.74%) in the non-MACE group (*P* = 0.040). The number of patients with multi-vessel disease in the MACE group was 19 (57.58%), which was higher than 119 patients (38.14%) in the non-MACE group (*P* = 0.030. The average time from symptom onset to hospital admission in the MACE group was 18.53 h, which did not differ significantly from 19.10 h in the non-MACE group (*P* = 0.179). Regarding disease type, 17 patients in the MACE group had ST-segment elevation AMI (STEMI), and 16 had non-ST-segment elevation AMI (NSTEMI), which showed no significant difference compared to 172 and 140 cases in the non-MACE group, respectively (*P* = 0.692). In the MACE group, 25 patients were classified as having Type 1 AMI and 8 as Type 2 AMI, compared with 235 and 77 cases, respectively, in the non-MACE group, showing no significant difference (*P* = 0.956). Regarding infarction location, 6 patients in the MACE group had anterior wall infarction, 14 had posterior wall infarction, and 13 had inferior wall infarction, compared with 73, 111, and 128 cases, respectively, in the non-MACE group, also with no significant difference (*P* = 0.684, Table 2).

**Table 2:** Comparison of disease-related characteristics between the MACE and non-MACE groups.

Disease-related characteristics		MACE group (*n* = 33)	Non-MACE group (*n* = 312)	χ^2^/*t*	*P*
Cardiac function class	Ⅰ-Ⅱ	12	172	4.222	0.040
	Ⅲ-Ⅳ	21	140		
Number of diseased vessels	Single-vessel	14	193	4.697	0.030
	Multi-vessel	19	119		
Time from onset to admission ($\bar{\chi }$ ± s, h)		18.53 ± 2.02	19.10 ± 2.34	1.347	0.179
AMI classification	Type 1	25	235	0.003	0.956
	Type 2	8	77		
Location of infarction	Anterior wall	6	73	0.761	0.684
	Posterior wall	14	111		
	Inferior wall	13	128		

### Comparison of laboratory indicators between the MACE and non-MACE groups

**Figure 1:**
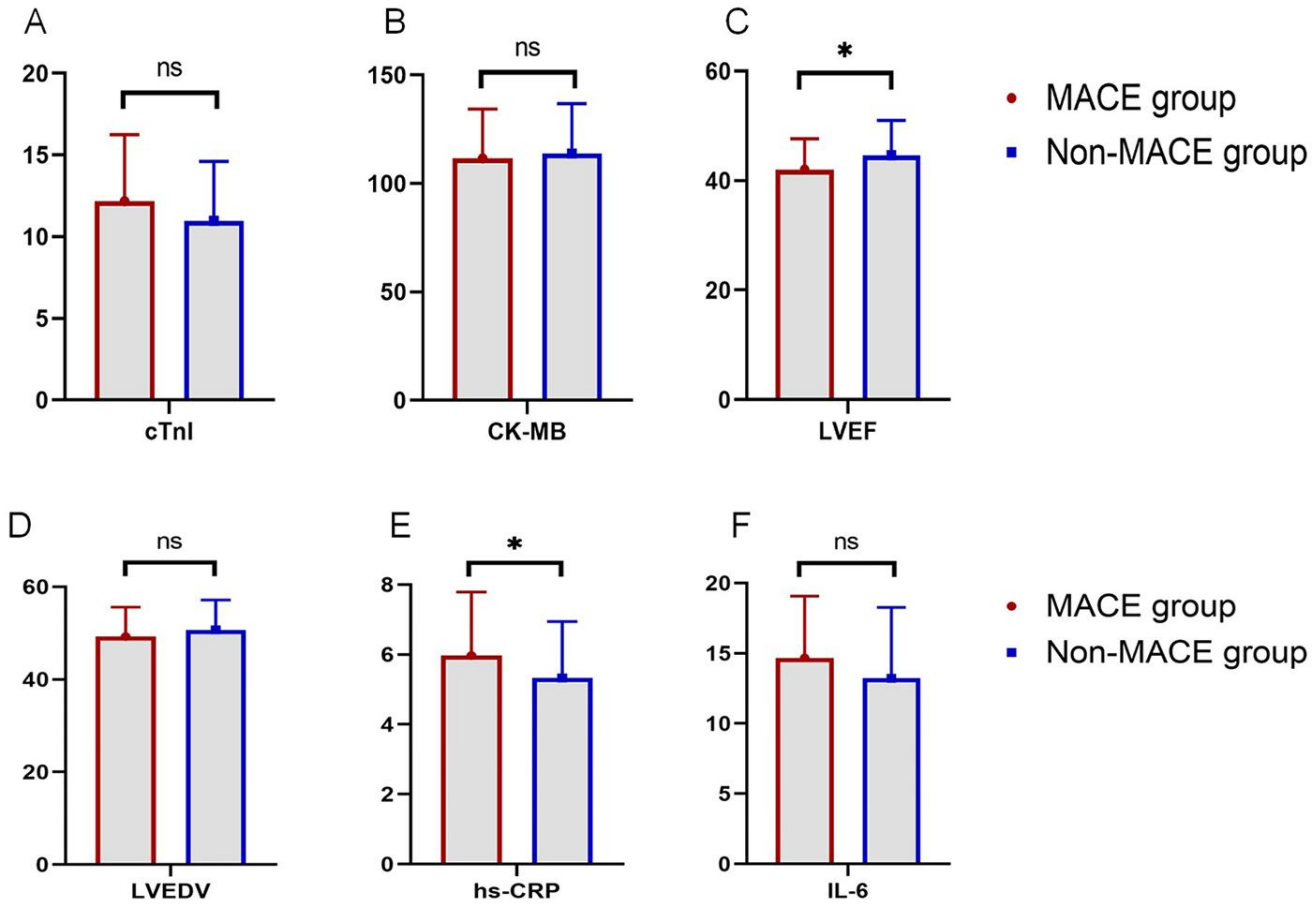
Comparison of laboratory indicators between the MACE and non-MACE groups (ns, not significant, * indicates *P* < 0.05). **(A)** Comparison of cTnI levels between groups. **(B)** Comparison of CK-MB levels between groups. **(C)** Comparison of LVEF between groups. **(D)** Comparison of LVEDV between groups. **(E)** Comparison of hs-CRP levels between groups. **(F)** Comparison of IL-6 levels between groups.

To evaluate laboratory parameters in the MACE and non-MACE groups, the following results were observed: The mean LVEF in the MACE group was 42.04%, significantly lower than 44.61% in the non-MACE group (*P* = 0.027). The level of hs-CRP in the MACE group was 5.97 mg/L, significantly higher than 5.33 mg/L in the non-MACE group (*P* = 0.035). However, no significant differences were found between the two groups in cTnI levels (12.18 vs. 10.97 ng/ml, *P* = 0.073), creatine kinase-MB (CK-MB) (111.53 vs. 113.82 U/L, *P* = 0.588), LVEDV (49.26 vs. 50.71 ml, *P* = 0.221), or IL-6 (14.68 vs. 13.22 pg/ml, *P* = 0.111, Figure 1).

### Comparison of quality of life between the MACE and Non-MACE groups

**Figure 2:**
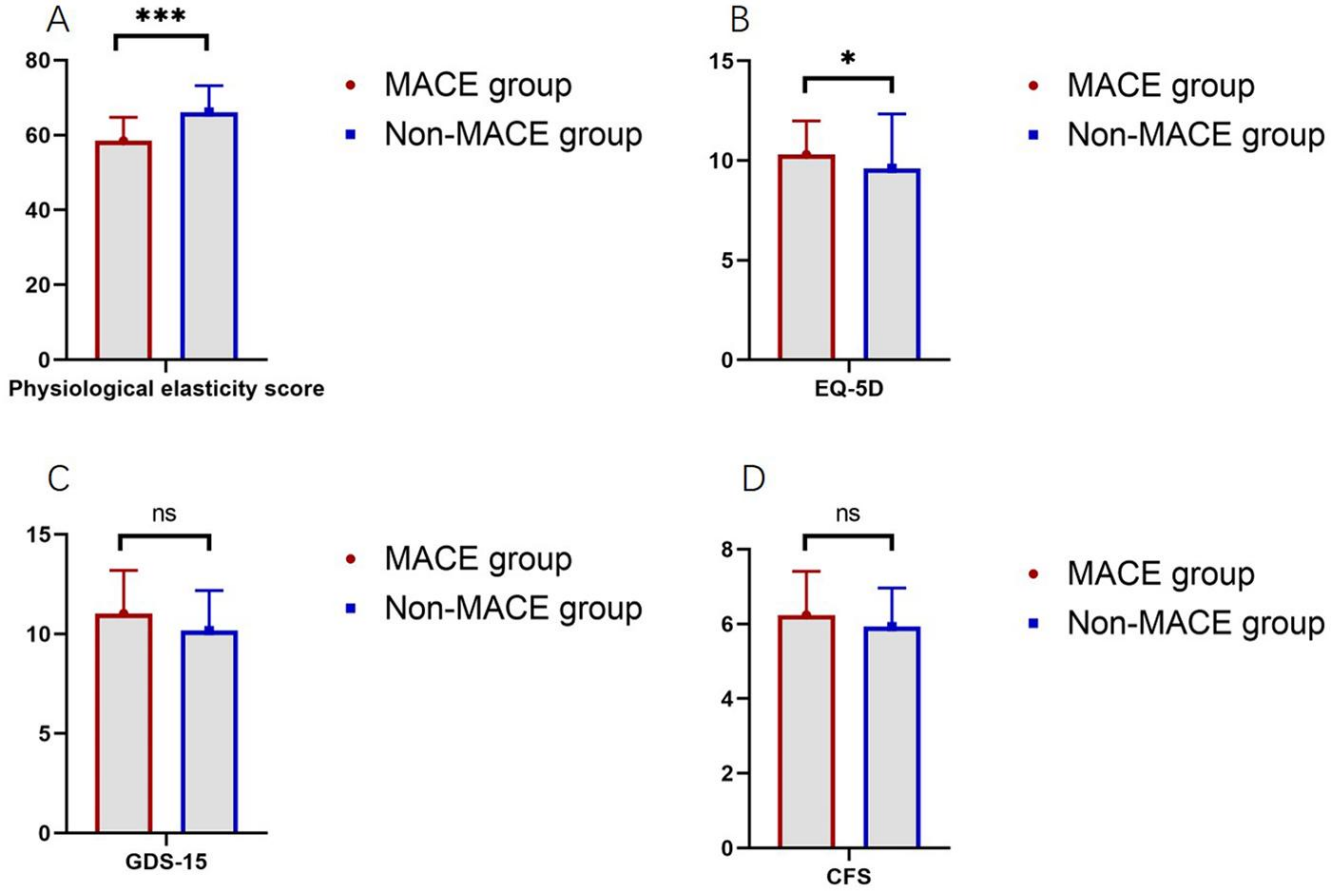
Comparison of quality of life scores between the MACE and non-MACE groups (ns, not significant, * indicates *P* < 0.05, *** indicates *P* < 0.001). **(A)** Comparison of physiological elasticity score between groups. **(B)** Comparison of EQ-5D between groups. **(C)** Comparison of GDS-15 between groups. **(D)** Comparison of CFS between groups.

To assess the quality of life among patients in both groups, health-related quality of life, depression, and frailty scores were analyzed. The physiological resilience score in the MACE group was 58.48, significantly lower than 66.15 in the non-MACE group (*P* < 0.001). The EQ-5D score in the MACE group was 10.31, which was significantly higher than 9.61 in the non-MACE group (*P* = 0.022). No significant difference was observed in the GDS-15 scores between the two groups (11.03 vs. 10.17, *P* = 0.153). The CFS scores were also comparable between groups (6.24 vs. 5.93, *P* = 0.100, Figure 2).

### Comparison of immune resilience indicators between the MACE and non-MACE groups

To evaluate systemic immune status, immune resilience indicators were compared between groups. The average LYM count in the MACE group was 1.70 × 10⁹/L, significantly lower than 1.93 × 10⁹/L in the non-MACE group (*P* = 0.044). The SII was 365.41 in the MACE group, significantly higher than 350.98 in the non-MACE group (*P* < 0.001). The systemic inflammation response index (SIRI) was markedly elevated in the MACE group (1.62) compared to the non-MACE group (1.25) (*P* < 0.001). However, no significant differences were observed in PLT (211.06 vs. 208.45 × 10⁹/L, *P* = 0.275), NEU (5.37 vs. 5.59 × 10⁹/L, *P* = 0.177), MON (0.69 vs. 0.64 × 10⁹/L, *P* = 0.117), or MLR (0.41 vs. 0.45; *P* > 0.05, Table 3).

**Table 3:** Comparison of immune resilience indicators between the MACE and non-MACE groups ($\bar{\chi }$ ± s).

Immune resilience indicators	MACE group (*n* = 33)	Non-MACE group (*n* = 312)	*t*	*P*
PLT (×10^9^/L)	211.06 ± 13.40	208.45 ± 13.01	1.093	0.275
NEU (×10^9^/L)	5.37 ± 1.78	5.59 ± 1.80	0.668	0.504
LYM (×10^9^/L)	1.70 ± 0.55	1.93 ± 0.63	2.017	0.044
MON (×10^9^/L)	0.69 ± 0.22	0.64 ± 0.20	1.353	0.177
MLR	0.41 ± 0.13	0.45 ± 0.14	1.571	0.117
SII	365.41 ± 18.59	350.98 ± 16.43	4.736	<0.001
SIRI	1.62 ± 0.46	1.25 ± 0.33	5.872	<0.001

### LASSO regression

**Figure 3:**
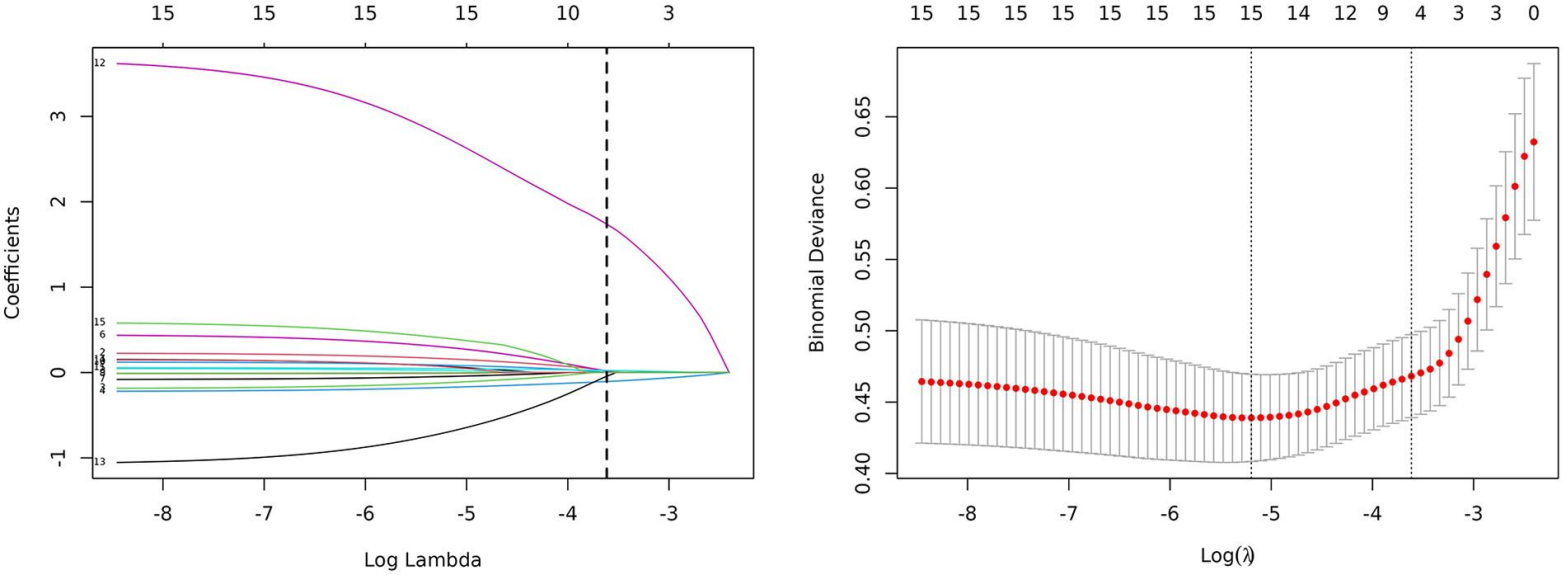
Clinical feature selection based on the LASSO regression model.

To identify predictors of adverse cardiac outcomes in patients with AMI. Least Absolute Shrinkage and Selection Operator (LASSO) regression was applied with adverse cardiac outcome as the dependent variable. The model was used to select variables from all statistically significant influencing factors. Ten-fold cross-validation was conducted to determine the optimal penalty parameter λ. As the penalty coefficient λ increased, the regression coefficients of the independent variables were gradually shrunk (Figure 3A). The λ value corresponding to the minimum cross-validation error (*λ* = 0.027) was selected as the optimal value (see Figure 3B). The results indicated that when *λ* = 0.027, the cross-validation error reached its minimum, suggesting that the model achieved an optimal balance between bias and variance. At this point, the penalty strength was moderate—avoiding overfitting caused by an excessively small λ and underfitting resulting from an overly large λ. Therefore, *λ* = 0.027 was determined as the optimal penalty coefficient. ① Univariate screening: Variables with *P* ≥ 0.1 (e.g., sex and age) were excluded from all potential predictors, leaving 15 variables for further analysis. ② Standardization: The 15 variables were standardized using *Z*-score normalization to eliminate dimensional effects. ③ LASSO coefficient shrinkage: Based on the optimal *λ* = 0.027, 8 variables were compressed to zero, and 7 variables with nonzero coefficients were finally retained.

### Multivariate logistic regression analysis of adverse cardiac outcomes in patients with AMI

The seven variables selected by the LASSO regression model were entered into a ***logistic*** proportional hazards regression model. Stepwise regression was used to identify independent risk and protective factors for adverse cardiac outcomes in AMI patients. The results showed that the physiological resilience score (OR = 0.812) was an independent protective factor for adverse cardiac outcomes in patients with AMI, whereas hs-CRP (OR = 1.622), SII (OR = 1.054), and SIRI (OR = 25.905) were identified as independent risk factors (Table 4).

**Table 4:** Multivariate logistic regression analysis of adverse cardiac outcomes in patients with AMI.

Variable	Regression coefficient	Standard error	*z*-value	*P*-value	OR	95% CI for OR
GDS-15	0.272	0.159	1.708	0.088	1.313	0.961–1.795
Physiological resilience score	−0.208	0.053	−3.895	<0.001	0.812	0.732–0.902
hs-CRP	0.484	0.219	2.205	0.027	1.622	1.055–2.492
cTnI	0.118	0.087	1.355	0.175	1.125	0.949–1.334
SII	0.053	0.022	2.403	0.016	1.054	1.010–1.101
SIRI	3.254	0.792	3.577	<0.001	25.905	5.491–122.213
LYM	−0.490	0.489	−1.003	0.316	0.613	0.235–1.596

### Model calibration and validation

**Figure 4:**
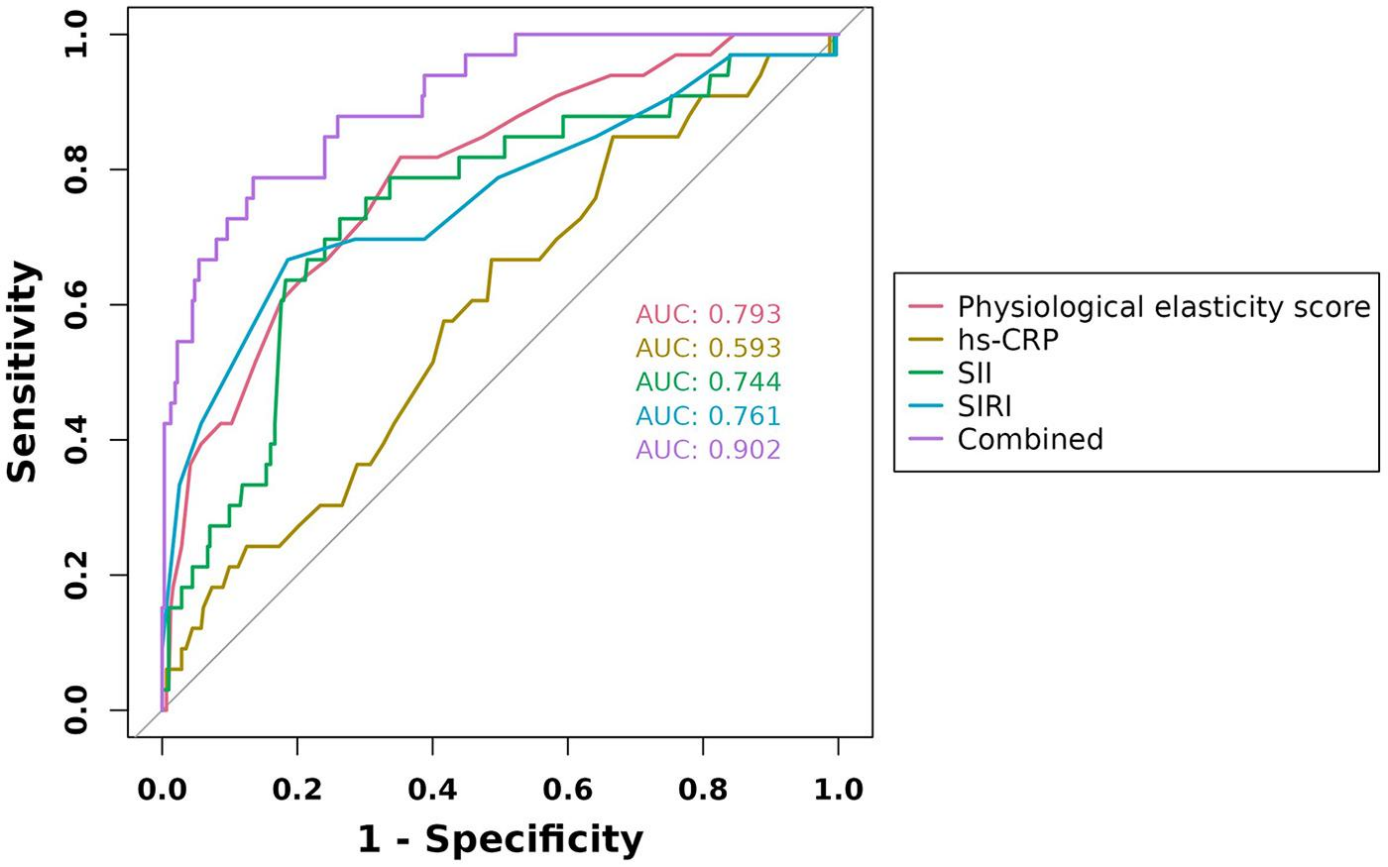
ROC curves of physiological elasticity score, hs-CRP, SII, and SIRI in predicting adverse cardiac outcomes in AMI patients.

**Figure 5:**
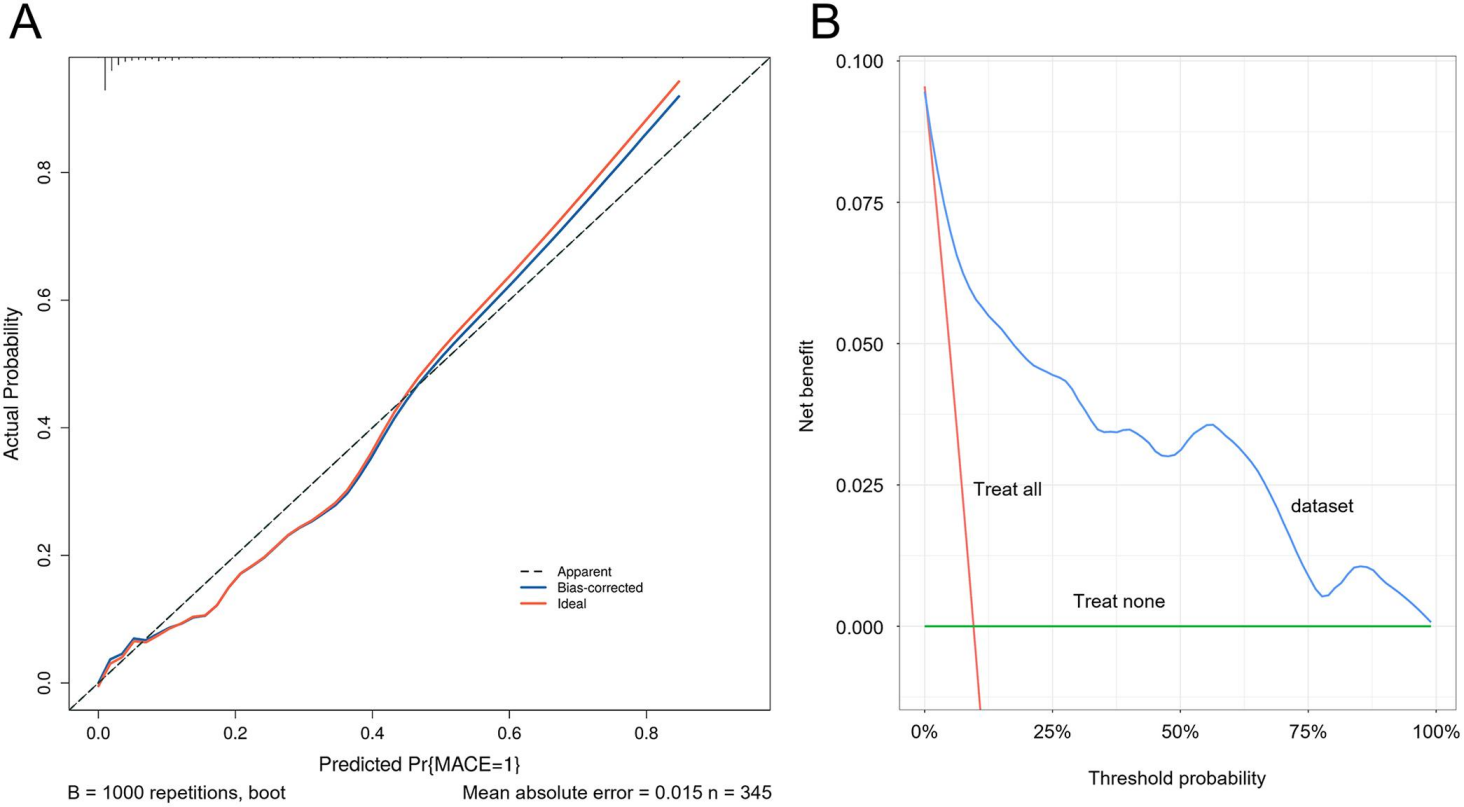
Calibration curve and decision curve of the predictive model. **(A)** Calibration curve of the predictive model. **(B)** Decision curve of the predictive model.

ROC curves were generated for the four variables—physiological resilience score, hs-CRP, SII, and SIRI—to evaluate their predictive value for adverse cardiac outcomes in patients with AMI. The results showed that the AUC values for physiological resilience score, hs-CRP, SII, and SIRI were 0.793, 0.593, 0.744, and 0.761, respectively. When combined, the AUC reached 0.902, indicating that the integrated model had a higher predictive accuracy for adverse cardiac outcomes in AMI patients (Figure 4). Model validation was performed using the Bootstrap method with 1,000 iterations, yielding a Nagelkerke R^2^ = 0.543, suggesting strong explanatory power for the dependent variable and demonstrating good model calibration (Figure 5A). Additionally, the Decision Curve Analysis (DCA) curve was higher than the two extreme reference curves, indicating a greater net clinical benefit of the model's predictive factors (Figure 5B).

## Discussion

PCI is the preferred treatment strategy for AMI, offering rapid revascularization of coronary arteries compared to conventional thrombolysis. It effectively restores blood supply to ischemic myocardium, improves postoperative cardiac function and prognosis, and significantly reduces mortality rates. However, clinical practice has revealed (14) that some patients still experience suboptimal outcomes after PCI, which contributes to increased mortality. One study reported a 30-day incidence of MACE of 9.65% in patients presenting with acute chest pain (15). In the present study, 345 AMI patients were included, among whom 33 experienced MACE, resulting in an incidence rate of 9.57%, which is consistent with prior clinical findings and confirms the presence of a substantial risk for adverse cardiac outcomes post-PCI in AMI patients. Nevertheless, the occurrence of MACE following PCI in AMI patients is influenced by multiple factors, and early identification and timely intervention are of great significance in improving patient prognosis.

In recent years, numerous studies have demonstrated that physiological resilience plays a critical role in clinical outcomes such as mortality and functional recovery. It has been suggested as a valuable tool for early identification of high-risk individuals, risk stratification, and prediction of adverse outcomes (16, 17). Furthermore, it has been reported that physiological resilience is closely associated with health-related quality of life in patients after AMI (18). In this study, an in-depth analysis revealed that the physiological resilience score in the MACE group was significantly lower than in the non-MACE group, while the EQ-5D score was higher. This finding is consistent with previous clinical studies, suggesting that the occurrence of MACE is associated with a marked reduction in physiological resilience and a substantial decline in quality of life among AMI patients. It was found that myocardial necrosis is inherent in AMI patients, and the occurrence of MACE further deteriorates cardiac function, severely impairing the heart's physiological resilience and its ability to pump blood effectively. This insufficiency manifests in symptoms such as dyspnea and fatigue, ultimately diminishing the patient's quality of life (19). Additionally, MACE is frequently accompanied by ventricular arrhythmias and atrioventricular conduction block, which directly disrupt the heart's rhythm and contractility, reducing cardiac output, further impairing physiological resilience, and lowering quality of life. Moreover, the components of MACE—including all-cause death, non-fatal AMI, arrhythmia, rehospitalization due to heart failure, revascularization, stroke, and major bleeding events—can each cause structural damage to the heart, leading to a rapid decline in cardiac function, loss of physiological resilience, and serious impairment of quality of life.

LVEF is an important indicator for evaluating cardiac systolic function. A decrease in LVEF suggests cardiac insufficiency or poor prognosis. hs-CRP is a sensitive marker of chronic low-grade inflammation, and its elevation is associated with worsening inflammatory status. It has been reported that the occurrence of MACE in AMI patients is positively correlated with hs-CRP and negatively correlated with LVEF (20). Consistent with these observations, the present study yielded similar findings, showing that LVEF was lower in the MACE group than in the non-MACE group, whereas hs-CRP levels were significantly higher in the MACE group. This suggests that AMI patients who develop MACE after PCI exhibit more severely impaired cardiac function and a heightened inflammatory status. Our analysis indicates that the occurrence of MACE in AMI is associated with a marked expansion of the area of myocardial necrosis, which may further increase the risk of arrhythmias and heart failure, profoundly impair myocardial contractility, and directly reduce left ventricular pump function, thereby lowering LVEF. Moreover, if myocardial stunning in the infarcted region is not alleviated after PCI, the resulting reduction in contractile efficiency may also contribute to a further decline in LVEF (21). In addition, the pathological basis of AMI involves atherosclerotic plaque rupture. As an acute-phase reactant, hs-CRP is synthesized in the liver under the stimulation of cytokines such as IL-6. Its elevated level directly reflects the intensity of the inflammatory response following plaque rupture. Simultaneously, myocardial cell necrosis often occurs in these patients, leading to the release of damage-associated molecular patterns (DAMPs) that activate Toll-like receptor signaling pathways, promote the secretion of inflammatory cytokines, and stimulate the liver to produce hs-CRP. During myocardial ischemia-reperfusion, large amounts of reactive oxygen species (ROS) are generated, which oxidize low-density lipoproteins and stimulate endothelial cells and macrophages, resulting in the secretion of inflammatory mediators and a direct increase in hs-CRP levels (22, 23). Although LVEF and hs-CRP have certain clinical value in predicting MACE in AMI patients, they cannot fully reflect the degree of myocardial injury, are easily influenced by other factors, and lack specificity, which limits their predictive efficacy.

With the deepening of clinical research, it has been found that single inflammatory markers such as hs-CRP primarily reflect the intensity of the inflammatory response but fail to accurately predict patient prognosis (24). Immune resilience indicators, on the other hand, have significant clinical importance for risk assessment in AMI patients. By integrating various peripheral blood cell counts, these indicators reflect both the severity of inflammation and the balance of the immune system, thereby providing more precise evidence for prognosis evaluation. At present, blood cell counts are often used clinically for such evaluations. For example, multiple studies have proposed that leukocyte count and NEU are independent risk factors for MACE (25), while other reports suggest that a decrease in PLT levels is associated with MACE (26). In this study, novel immune resilience indicators were developed by integrating various blood cell counts. The results revealed significant differences between the MACE and non-MACE groups in LYM, SII, and SIRI, whereas PLT, NEU, MON, and MLR showed no significant differences between the two groups. This conclusion differs slightly from previous clinical studies, which may be attributed to population heterogeneity, study design, and follow-up duration. After the onset of AMI, patients enter a stress state accompanied by an activated inflammatory response. PLT, as a crucial component of the immune system, plays an important role during inflammation and infection. The occurrence of MACE suggests further myocardial injury and intensified inflammation, resulting in extensive PLT consumption and even inhibition of its proliferation and differentiation, ultimately leading to reduced PLT counts (27). SII and SIRI are novel immune resilience indicators. SII, which is calculated based on PLT, NEU, and LYM, mainly reflects the body's immune-inflammatory status. It was found that when MACE occurs in AMI patients, the inflammatory response intensifies, NEU are activated and accumulate at myocardial injury sites, promoting the production of various inflammatory mediators and exacerbating myocardial damage. Meanwhile, PLT count and activity change during inflammation and thrombosis, and LYM levels decrease significantly, collectively leading to an increase in SII (28). SIRI, calculated from NEU, MON, and LYM, reflects the systemic inflammatory status of patients. When MACE occurs, the cytokine network becomes imbalanced, with excessive secretion of pro-inflammatory cytokines and insufficient anti-inflammatory factors. This imbalance results in the activation and recruitment of NEU and MON, while LYM levels decline, causing a marked elevation in SIRI (29).

This study demonstrated that physiological resilience score, hs-CRP, SII, and SIRI are influencing factors for adverse cardiac outcomes in AMI patients. Furthermore, these indicators showed high predictive value for adverse cardiac outcomes. Since physiological and immune resilience are closely associated with adverse cardiac events, combining them to construct a physiological-immune resilience risk assessment model can improve prediction accuracy, facilitate early identification of high-risk patients, optimize clinical decision-making, and ultimately improve prognosis. Physiological resilience is not only associated with an individual's recovery ability but also influences long-term survival and quality of life. Therefore, assessing physiological resilience allows accurate prognosis prediction and provides guidance for individualized treatment strategies. In contrast, SII and SIRI, as novel inflammatory markers derived from peripheral blood cell counts, can reflect systemic immune and inflammatory status. Compared to single indicators, they offer more comprehensive information and thereby enhance the accuracy of predicting MACE in AMI patients (30).

Nevertheless, several limitations should be acknowledged. First, this study was conducted as a single-center investigation with a limited sample size. The risk model was established within the same institutional cohort without standardized internal or external multi-center validation, thereby making it difficult to exclude the risk of overfitting and potentially limiting the generalizability of the findings. Second, the absence of long-term follow-up after discharge restricts the ability to comprehensively evaluate patients' medium- and long-term prognoses, thus limiting the model's applicability in predicting extended outcomes. In this study, inflammatory biomarkers were sampled at different time points; however, because the systemic inflammatory response is dynamically influenced by AMI-related stress and PCI over time, these temporal differences may affect the stability and between-group comparability of systemic immune–inflammatory indices, thereby introducing potential bias into the interpretation of their associations with MACE. Moreover, the PRIFOR, EQ-5D, GDS-15, and CFS scales used in this study have been primarily validated in chronic or clinically stable populations, whereas our participants were patients with acute AMI, whose physiological and psychological states differ substantially from those of stable cohorts. As a result, these instruments may not fully or accurately capture the patients' true status in the acute phase, which could in turn bias the interpretation of the relationships between these measures and the occurrence of MACE. Moreover, the variables incorporated into the model may not have been sufficiently comprehensive, as several clinically relevant factors—such as Killip classification, renal function indicators, use of standardized therapeutic regimens, and coronary angiographic complexity—were not included. The omission of these variables may have weakened the robustness of the model and introduced bias into risk estimation. Although SIRI was identified as an independent risk factor, its notably high odds ratio and wide confidence interval suggest instability in estimation. In light of these considerations, future research should aim to integrate standardized data from multiple medical institutions and conduct independent external cohort validation to develop a dynamically updated risk prediction model with improved universality and accuracy. Additionally, longitudinal follow-up should be performed to assess the model's predictive value for long-term outcomes. Future studies should establish a standardized sampling protocol for inflammatory markers, with unified sampling time points to ensure synchronized measurements and improve between-group comparability, thereby minimizing the confounding effects of dynamic inflammatory changes. In addition, the applicability of these scales in patients with acute AMI should be validated and optimized, and acute phase–specific assessment tools may be introduced. The inclusion of key variables—such as Killip classification, renal function, medication adherence, and coronary lesion characteristics—alongside a psychosocial assessment module, would further refine the model by enhancing its bio-psycho-social comprehensiveness and improving the overall predictive performance.

In conclusion, patients with AMI remain at risk of adverse cardiac outcomes following PCI. This risk may be related to factors such as physiological resilience score, hs-CRP, SII, and SIRI. The combined use of these indicators for predicting 30-day MACE in AMI patients demonstrates high value. Therefore, early clinical interventions based on these indicators should be implemented to reduce the incidence of adverse cardiac outcomes and improve patient prognosis.

## Data Availability

The original contributions presented in the study are included in the article/Supplementary Material, further inquiries can be directed to the corresponding author.
